# Prevalence of sexually transmitted infections and associated factors among the University of Gondar students, Northwest Ethiopia: a cross-sectional study

**DOI:** 10.1186/s12978-019-0815-5

**Published:** 2019-11-08

**Authors:** Belayneh Ayanaw Kassie, Hedja Yenus, Resom Berhe, Eskeziaw Abebe Kassahun

**Affiliations:** 1 Department of Midwifery, College of Medicine and Health Sciences, University of Gondar, Gondar, Ethiopia; 2 Department of Reproductive Health, Institute of Public Health, University of Gondar, Gondar, Ethiopia; 3 Department of Health Education and Promotion, Institute of Public Health, University of Gondar, Gondar, Ethiopia; 4 Department of Midwifery, College of Health Sciences, Woldia University, Woldia, Ethiopia

**Keywords:** Sexually transmitted infections, University, Students, Gondar,Ethiopia

## Abstract

**Introduction:**

Globally, sexually transmitted infections (STIs) remain a major public health problem. University students tend to practices sex which predisposes them to sexually transmitted infections, unwanted pregnancies, and unsafe abortions due to their freedom from families. Therefore, the study aimed to assess the prevalence of sexually transmitted infections and associated factors among the University of Gondar students, northwest Ethiopia.

**Methods:**

An institution based cross-sectional study was conducted on 845 the University of Gondar students selected using the multistage sampling technique from March 7–10, 2016. The data were collected using a structured, pre-tested self-administered questionnaire. Bivariate and multivariable logistic regression analyses were employed through SPSS version 20 to identify factors associated with sexually transmitted infections. Odds ratio with a 95% confidence interval was computed to determine the level of association. In the multivariable analysis, variables with *p*-value less than 5% were considered as statistically significant association between covariates and sexually transmitted infections.

**Result:**

Sexually transmitted infections among university students were found to be 18.20% (95%CI,15.40,20.80). Previous history of sexually transmitted infections (AOR = 2.1; 95%CI: 1.04, 4.38), multiple sexual partners in life (AOR = 2.7; 95%CI:1.70, 4.40), not use of condoms during sexual intercourses (AOR = 2.4; 95%CI:1.50,3.75) and poor knowledge of sexually transmitted infections (AOR = 3.3; 95%CI:1.09,5.32) were significantly associated with sexually transmitted infections.

**Conclusion:**

The prevalence of STIs was high among university students. The previous history of sexually transmitted infections, multiple sexual partners, not using condoms during sexual intercourse and poor knowledge of sexually transmitted infections were found to be associated with the infections. Opening and strengthen reproductive health centers on the campuses, popularizing sexual, and reproductive health information and education, particularly on STI modes of transmission, prevention, and health-seeking behaviors, and providing information on accessing of condoms is recommended to reduce sexually transmitted infections.

## Plain English summary

Sexually transmitted infections (STIs) are a variety of clinical syndromes caused by pathogens that can be acquired and transmitted through sexual contact and remain a major public health problem. Sexually transmitted infections predispose to major health consequence such as infertility, certain cancers, and other chronic disease occurs year after the initial infection. The burden of STIs varies from area to area in the world particularly higher in low-income countries including Ethiopia. There has been limited information on the prevalence and associated factors of sexually transmitted infections among university students in Ethiopia including the study area. Therefore, this study aimed to assess the prevalence of sexually transmitted infections and its associated factors among the University of Gondar students.

A total of 803 students were included of which, 491(61.1%) of students ever practiced sex. Moreover, 18.2% of students were developed sexually transmitted infections. The previous history of sexually transmitted infections, multiple sexual partners in life, not the use of condoms during sexual intercourse and poor knowledge of sexually transmitted infections were significantly and independently associated with sexually transmitted infections. Therefore, improving student’s awareness of the risk factors and implications of sexually transmitted infection is crucial to overcome the burden.

## Background

Sexually transmitted infections (STIs) are a variety of clinical syndromes caused by pathogens that can be acquired and transmitted through sexual contact. There are over 30 bacterial, viral, and parasitic pathogens that have been identified to date to be transmitted sexually [1]. Sexually transmitted infections are a major public health problem worldwide that cause of acute illness, long-term complication, infertility, medical as well as psychological consequences and death [2]. Moreover, STIs facilitate the spread of the human immunodeficiency virus (HIV) [3]. In 2012, 498.9 and 92.6 million new cases of STIs occurred on the globe and in Africa, respectively. Thus, on average, about 1.4 million people are infected with STIs every day [4]. In Ethiopia, the highest reported rates of STIs are found among 15–24-year old, while about half of all of the people infected with HIV and 60% of all new HIV infections are also in that age group [5].

Because young people are at high risk for risky behaviors and low use of preventive mechanisms and/or services in developing nations, STIs were common sexual and reproductive health problems. Adolescents and young adults catch the highest rates of curable STIs, and 1 in 20 adolescents acquire new STIs each year [6]. Young people, especially those who are unlikely to have access to quality health care services, such as university students are at high risk of STIs [7]. About 80–90% of the global burden of STIs which found in the developing world where there is limited and/or no access to diagnostics of STIs [8].

Adolescents and the youth, in general, tend to experiment risky behavior due to their new freedom at boarding institutions, liberty from familiarized community, parents or guardians and teachers in secondary schools. University students are categorized under the most at-risk population segment (MARPS) due to their inclination to be engaged in risky sexual behavior and their poor sense of vulnerability [9–14]. Despite this, youth have not traditionally been considered a health priority since they have lower morbidity and mortality rates than older and younger age groups [15].

The national HIV/AIDS policy of Ethiopia identifies STIs prevention and control as one of the strategies to prevent and control HIV/AIDS [16]. Despite the large scale-up of health care investment for the prevention and treatment of STIs in Ethiopia [17], the prevalence among the Ethiopian youth rose from 1.15% in 2005 to 4% in 2011 [18, 19]. This continued increase in the prevalence of STIs among the youth may have a significant impact on the next fate of the country since the youth constitute the bulk of the future workforce [20]. In spite of the continued increase in the prevalence of STIs in the country, relatively little epidemiological research has been carried out on the prevalence and associated risk factors of STIs [21]. Hence, quantifying the burden of these infections and identifying factors among students of higher institutions is important for designing an effective intervention and allocating resources. Therefore, this study aimed to assess the prevalence of STIs and its associated factors among the University of Gondar regular undergraduate students.

## Methods

### Study design and setting

An institution based cross-sectional study was conducted from March 7–18 / 2016 at the University of Gondar, located in Gondar town, 748 km from Addis Ababa, the capital of Ethiopia. There were 32,962 students enrolled in different programs. There are three student clinics and one teaching and referral hospital providing STI diagnosis and treatment services to students.

### Sample size and sampling procedures

All students attended the University of Gondar in the regular undergraduate program during the study period were eligible for the study. The sample size was calculated using the single population proportion formula by considering the prevalence of STIs among university students as 19.5% [22], a 95% confidence interval (CI), and a 4% margin of error. By adding a 10% non-response rate and a design effect of 2, the final sample was 845 students. A multistage sampling technique was used to select participants. Considering 30% rule of thumb and simple random sampling technique with proportional allocation, 13 departments were selected. The final samples were selected from the specified departments using the systematic random sampling technique.

### Data collection tool and procedures

Data was collected using a structured self-administered questionnaire prepared in English. To maintain the privacy of participants, seats were arranged far apart. The questionnaire was pre-tested on 42 Debre-Tabor University students outside the study area. Three days of intensive training was given to 10 data collectors selected from the University of Gondar, Institute of Public Health, before data collection. The data were collected on different variables such as age, sex, religion, marital status, ethnicity, religious involvement, year of study, family residence, monthly pocket money, age at first sexual initiation, number of sexual partners, unprotected sex, sex with commercial sex workers (CSWs)**,** knowledge on STIs, substance use, watch/read, pornography, peer pressure to had sex and previous history of STIs. A male student was considered as STIs positive if he reported one or more of the following syndromes: a history of Genital ulcer or sores, urethral discharge, scrotal swelling, inguinal bubo syndromes in the past 12 months prior to data collection. A female student was considered as STIs positive, if she reported one or more of the following syndromes: abnormal vaginal discharge, genital ulcer or sores, lower abdominal pain syndromes in the past 12 months prior to data collection. Knowledge of STIs was assessed using different questions which contained having information on STIs, prevention, ways of transmission, sign and symptoms and possible complications of STIs. Students who scored the mean and above in knowledge assessment questions were considered having good knowledge of STIs.

### Data processing and analysis

Data were checked, coded, and entered into EpiData version 3.1 and exported to SPSS version 20 statistical software for further analysis. Descriptive statistics were used to characterize participants using different variables. Both bivariate and multivariable logistic regression analyses were done to identify factors associated with STIs. Variables with P- value ≤0.2 in the bivariate logistic regression were fitted into the multivariable logistic regression analysis. Prior to the multivariable analysis, multicollinearity diagnostic was performed, and there was no significant interaction between independent variables. Adjusted Odds Ratio (AOR) with a 95% Confidence Interval (CI) was calculated to determine the presence and strength of association. In the multivariable analysis, a variable with a *p*-value less than 0.05 was considered statistically significant.

## Result

### Socio-demographic and family-related characteristics

A total of 803 university students participated in the study with a response rate of 95%. The median age of the participants was 21 years (IQR ± of 2 years). Of the participants, 52.9% were male,58.3% Orthodox Christian, and 87.9% were single. The majority (96.9%) of the students were boarding, and 87.3% received pocket money from families and relatives (Table 1).

**Table 1:** Socio-demographic characteristics of the University of Gondar regular undergraduate students, northwest Ethiopia, 2016 (*n* = 803)

Variables	Frequency	Percentage	
Sex			
Male	425	52.9	
Female	378	47.1	
Age			
15–19	98	12.2	
20–24	675	84.1	
25–29	26	3.2	
30–34	4	0.5	
Marital status			
Single	725	90.3	
Married	67	8.3	
Others^a^	11	1.4	
Year of Study			
1st Year	194	24.2	
2nd Year	190	23.7	
3rd Year	242	30.1	
4th Year and/or above	177	22.0	
Ethnicity			
Amhara	551	68.6	
Oromo	84	10.5	
Tigre	57	7.1	
Somali	34	4.2	
SNNPR	42	5.2	
Others^b^	35	4.4	
Residence			
Urban	415	51.7	
Rural	388	48.3	
Religion			
Orthodox	468	58.3	
Muslim	154	19.2	
Protestant	91	11.3	
Catholic	77	9.6	
Adventist	13	1.6	
Religious service attendance			
Every day	303	37.7	
At least once a week	328	40.8	
At least once a month	105	13.1	
At least once a year	61	7.6	
Never attend	6	0.8	

^a^Divorced, widowed ^b^Benishangul Gumuz, Afar

### Behavioral characteristics

About 60% of students drank alcohol, 11.6% chewed khat, 6.1% smoked shisha, and 75.5% seen or read pornography **(**Table 2**).**

**Table 2:** Behavioral characteristics of the University of Gondar regular undergraduate students, northwest Ethiopia, 2016 (*n* = 803)

Variables	Frequency	Percentage
Alcohol drinking		
Yes	409	50.9
No	394	49.1
Khat Chewing		
Yes	93	11.6
No	710	88.4
Smoke shisha		
Yes	49	6.1
No	754	93.9
Ever watch or read pornographic materials		
Yes	443	55.2
No	360	44.8
Watch/read pornographic materials in the last 12 months (*n* = 443)		
Yes	333	75.2
No	110	24.8
Visited night club		
Yes	299	37.2
No	504	62.8

### Sexual characteristics

About 61.1% of participants, had ever sex, of which 73.5% had sexual intercourse in the last 12 months. The mean age at first sexual intercourse was 18.8 (SD + 1.9) years. Furthermore, 42.8% of the students started a sexual activity after they joined the University. Of the sexually active respondents, 23.6% were initiated their first sex before the age of 18 years **(**Table 3**)**.

**Table 3:** Sexual characteristics of the University of Gondar regular undergraduate students, northwest Ethiopia, 2016 (*n* = 803)

Variables	Frequency	Percentage
Ever had sex (*n* = 803)		
Yes	491	61.1
No	312	38.9
Reason for fist sex (*n* = 491)		
Love	335	68.2
Peer pressure	71	14.5
To get money/benefits	36	7.3
Got married	44	5.5
Other^a^	5	1.0
First sexual partner (*n* = 491)		
Boy/girlfriend	350	71.3
Teacher	22	4.5
Casual partner	48	9.8
Husband or wife	44	9.0
Family member	8	1.6
Other^b^	19	3.8
Condom used during first sex (*n* = 491)		
Yes	210	42.8
No	281	57.2
Number of lifetime sexual partners (*n* = 491)		
One	225	45.8
Two and above	266	54.2
Sex under the influence of Alcohol (*n* = 409)		
Yes	128	31.3
No	281	68.7
Sex under the influence of Khat(*n* = 93)		
Yes	34	36.6
No	59	63.4
Sex under the influence of Shisha (*n* = 49)		
Yes	8	16.4
No	41	83.6

^a^Get drunk, raped ^b^CSWs, violent/rape, family servant

### Knowledge of sexually transmitted infections

Almost all students (98%) were ever heard about STIs. The majority of students (90.4%) reported that unprotected sex was the major mode of transmission for STIs. Moreover, more than half (55.3%) of students had good knowledge of STIs.

### Prevalence of sexually transmitted infections

**Fig. 1:**
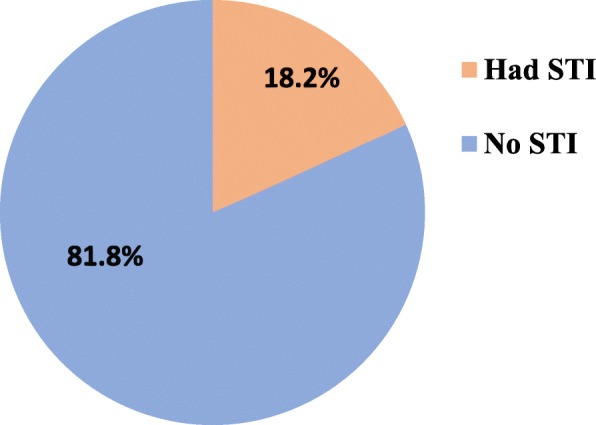
Prevalence of sexually transmitted infections among students in the last 12 months, the University of Gondar, northwest Ethiopia, 2016

**Fig. 2:**
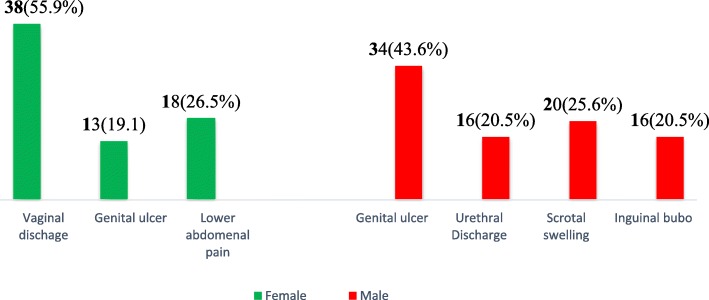
Syndromes of sexually transmitted infections reported by students, the University of Gondar, northwest Ethiopia, 2016

The overall prevalence of sexually transmitted infections at the University of Gondar was 18.2% (95% CI, 15.4, 20.8) in the past 12 months. About half (46.6%) female students were known to be infected **(**Fig. 1**).** In the last 12 months, genital ulcer and vaginal discharge were the most prevalent syndromes reported by 43.6 and 55.9% of the male and female students who had signs and symptoms of STIs, respectively **(**Fig. 2**).**

### Factors associated with sexually transmitted infections

Both bivariate and multivariable logistic regression analyses were done to see the effect of the selected characteristics on STIs. As presented in Table 4, peer pressure, viewed or read pornographic materials, chewing khat, drinking alcohol, the previous history of STIs, multiple lifetime sexual partners, no use of condoms and poor knowledge of STIs were found to have an association with STIs in the bivariate analyses at *p*-value less than 0.2. In the multivariable analyses, previous history of STIs, multiple lifetime sexual partners, no use condoms and poor knowledge of STIs were significantly associated with developing sexually transmitted infections.

**Table 4:** Bivariate and multivariable logistic regression analyses for factors associated with sexually transmitted infections among the University of Gondar students, northwest Ethiopia, 2016

Variables	Sexually transmitted infections		COR (95%CI)	AOR (95%CI)
	Yes	No		
Watching/reading pornography				
Yes	107	336	2.6 (1.76,4.00)	1.5 (0.93,2.50)
No	39	321	1.00	1.00
Peer pressure to had sex				
Yes	94	247	3 (2.06,4.36)	1.3 (0.82,2.08)
No	52	410	1.00	1.00
Ever had STI (in life)				
Yes	25	20	3.36 (1.80,6.27)	2.1 (1.04,4.38) *
No	121	325	1.00	1.00
Drink alcohol				
Yes	96	313	2.1 (1.45,3.07)	0.85 (0.52,1.40)
No	50	344	1.00	1.00
Chew khat				
Yes	37	56	3.6 (2.30,5.80)	1.2 (0.67,2.10)
No	109	601	1.00	1.00
Number of Sexual partners in life				
Two and above	100	166	2.3 (1.56,3.50)	2.7 (1.70,4.40) **
One	46	179	1.00	1.00
Ever used condom				
No	76	125	1.9 (1.3,2.80)	2.4 (1.50,3.75) **
Yes	70	220	1.00	1.00
Knowledge of STIS				
Poor	103	256	3.75 (2.54,5.50)	3.3 (1.09,5.32) **
Good	43	401	1.00	1.00

Note: *(*p*-value < 0.05) and **(*p*-value < 0.01)

The odds of having STIs were two times higher among students who had the previous history of STIs (AOR = 2.1; 95% CI: 1.04,4.38) compare to no history of STIs. Students who didn’t use condoms had two times more chance of acquiring STIs than those students who had used (AOR = 2.4; 95% CI: 1.50:3.75). Likewise, the odds of developing STIs among students who had two or more sexual partners in life were 2.7 times (AOR = 2.7; 95% CI:1.70,4.40) higher as compared to those who had a single sexual partner. Moreover, students who had poor knowledge of STIs were about three times (AOR = 3.3; 95% CI: 1.09,5.32) higher odds of acquiring STIs as compared to the knowledgeable students **(**Table 4**)**.

## Discussion

Identifying of sexually transmitted infections and providing relevant information, and offering the necessary health services is a highly effective intervention in the prevention and control of STIs. The prevalence of STIs among university students in the last 12 months was 18.2% (95% CI; 15.4, 20.8%). The finding is comparable with those of studies conducted on the University students in Wolaita Sodo (19.5%) [22] and Addis Ababa (15.74%) [23]. But, this finding was slightly lower than those of other studies conducted on Debre Birhan University students (28%) [24] and female youth at Mekelle (21.3%) [25]. The possible difference from Mekelle study might be due to the difference in the study area and study subjects were selected from the health facilities where there was a high probability of finding suspected cases. Furthermore, the possible difference might be due to the differences in the study subjects. This study included both male and female while Mekelle study included female only. Due to that female’s anatomy can place at higher risk of sexually transmitted infections than males [26].

The finding from this study was higher when compared with Ethiopian Demography and Health Survey (EDHS) 2011 report, among the youth (4%) [19], Bonga college students (13.9%) [27], Bahir Dar University students (6.4%) [28] and high school students in Gondar (10.7%) [29]. The possible difference from the EDHS report might be due to that EDHS survey was a population-based survey which included youth who are living with, and are under the control of the family. The data collection methods might also account for the variation. Likewise, in the Bonga and Gondar, the variation could be due to the differences in age and the student’s lifestyle. Residing and living with families and relatives helps the parent or relatives to monitor their children and improve decision-making capacity on risky health behaviors [13, 14]. Besides, the Gondar and Bahir Dar research included only female students.

In this study, 61.1% of the study subjects reported to had sex, of these 54.2% have multiple sexual partners in their life. Of the sexually active study subjects in the previous year before the survey, 23.6% were initiated sex before the age of 18 years and 31.3% had sex under the influence of substance. The fact is that risky sexual behaviors such as having multiple sexual partners, having sex before 18 years old and sex under the influence of substance can expose individuals for the risk of contracting sexually transmitted infections (STIs) [30].

Large number (75%) of students were exposed for pornography. Exposing to pornographic materials could alter the normal sexual desire and care taking of exposing to sexually transmitted infections [31, 32].

Having good knowledge of STIs is one of the protective factors for students to be aware of the modes of transmission, prevention methods and its complications which helps to take care of themselves from STIs. In this study, students with poor knowledge of STIs were more likely to develop STIs than students with good knowledge. This finding agrees with those of other studies conducted in Mekelle [25], Wolaita Sodo University [27], and Bahir Dar town [33].

Similarly, having multiple sexual partners is a known risk factor of sexual and reproductive health. Since university students come from different regions and towns with different cultures and values, they may be vulnerable to influences in their new environment as they live away from family or relatives with new friends and classmates. This may limit the student’s capability to defend themselves from peer pressure. Students could also be easily betrayed by monetary incentives from individuals [13, 14]. In this study, students who had two or more sexual partners in life were more likely to acquiring STIs than a student who had only one sexual partner. This is consistent with the various studies conducted at Mada Wulabu university [34], on Malawian youth [35], and at Bahir Dar [33].

Condom use is one of the methods of preventing the transmission of STIs [36]. Individuals who had never used condoms were significantly associated with STIs. Accordingly, the higher odds of STIs were observed among students who had never used condoms during sexual intercourse. This finding is supported by the previous studies in Debre Berhan [24]. This could be because of individuals who used condoms might have more access, information, and experiences in its appropriate use.

Having the previous history of STIs had a positive association with the development of STIs, that is, students who previously had STIs were more likely to develop STIs than students with no such history. This finding was supported by researches conducted from the United States [37] and Mekelle [25]. This might be due to relapse, untreated sexual partner, poor compliance with treatment, inappropriate treatment, and antimicrobial drug resistance.

Since sexual behavior and practice is private, intimate and sensitive, respondents may feel mortified or ashamed to report syndromes (may be subject to social desirability bias). To minimize the problem, we used a self-administered questionnaire and clearly informed participants about the purpose of the study and the confidentiality of information. Sexually transmitted infections were assessed only through students reports which approach may miss asymptomatic students and may misdiagnose signs/symptoms due to other problems similar to STIs might be the possible limitation of the study.

## Conclusion

In this study, the prevalence of self-reported STIs in the last 12 months among the University of Gondar regular undergraduate students was found to be high as compared to the national figure. Multiple sexual partners in life, previous history of STIs, not the use of condoms and poor knowledge of STIs were factors associated with STIs. Therefore, developing and strengthen reproductive health centers on the campuses, popularizing sexual and reproductive health information and education, particularly STI modes of transmission, prevention, health-seeking behaviors, and providing information and improving access to condom is recommended to reduce sexually transmitted infections.

## Data Availability

The datasets used and/or analyzed during the current study are available from the corresponding author on reasonable request.

## Abbreviations

AOR: Adjusted Odds Ratio
CI: Confidence Interval
COR: Crude Odds Ratio
IQR: Inter Quartile Range
SPSS: Statistical Package for Social Sciences
STI: Sexually Transmitted Infections
